# Beyond grasses: the potential benefits of studying silicon accumulation in non-grass species

**DOI:** 10.3389/fpls.2014.00376

**Published:** 2014-07-31

**Authors:** Ofir Katz

**Affiliations:** Department of Geography and Environmental Development, Ben-Gurion University of the NegevBe'er-Sheva, Israel

**Keywords:** Asteraceae, Fabaceae, Orchidaceae, Poaceae, phylogeny, phytolith, Rubiaceae, silicification

## Introduction

Silicification in angiosperms is a phenomenon that has attracted increasing attention in recent years. It is now widely acknowledged that silicification has many benefits to angiosperms (Richmond and Sussman, 2003; Ma and Yamaji, 2008; Epstein, 2009; Guntzer et al., 2012), and that it probably plays appreciable roles at the ecosystem and landscape levels as well (Cooke and Leishman, 2011; Reynolds et al., 2012; Schoelynck et al., 2014). High silica accumulating plant species are considered a major pool in the silicon cycle, affecting silicon fluxes and turnover rates (Conley, 2002; Falkowski et al., 2004; Derry et al., 2005; Sommer et al., 2006; Li et al., 2011; Carey and Fulweiler, 2012; Struyf and Conley, 2012; Vandevenne et al., 2013; Schoelynck et al., 2014). Indeed, recent studies have demonstrated the significant roles of plant silicification on the silicon cycle in grasslands (e.g., Melzer et al., 2012), freshwater and tidal ecosystems (e.g., Jacobs et al., 2013; Schoelynck et al., 2014) and forests (e.g., Farmer, 2005; Farmer et al., 2005; Cornelis et al., 2011). Furthermore, since silicate weathering is a CO_2_-consuming process, the effects of silica uptake and accumulation by plants on the silicon cycle may also influence the global carbon cycle (Street-Perrott and Barker, 2008).

Silica contents vary greatly among the angiosperms, with high concentrations occurring most commonly in the Poaceae (hereafter referred to as grasses) and other related monocotyledonous commelinid families (Hodson et al., 2005; Piperno, 2006) and aquatic macrophytes (Schoelynck et al., 2012). Due to the relatively high silica concentrations in grasses and to their high economic and ecological importance, silicification has been studied more frequently and more intensely in this family than in any other plant family. However, a better understanding of silicification in non-grass taxa can promote our understanding of the function and importance of silicon for plants. Unfortunately, studies of non-grass taxa are not only rarer, but also usually focus on a small number of taxa, mostly families and species that are silica-rich (e.g., Cucurbitaceae: Rogalla and Römheld, 2002), economically important (e.g., Fabaceae: Shen et al., 2010), or serve as model plant species (e.g., *Arabidopsis*: Fauteux et al., 2006), which are unlikely to be a fair representation of non-grass taxa. This means there is a significant gap in our knowledge of silicon processes, patterns and roles, given the fact that non-grass taxa, such as forest tree, are a major component of the silicon cycle (Farmer, 2005; Farmer et al., 2005; Cornelis et al., 2011). I shall therefore briefly discuss the variability of silicification among non-grass taxa, and how it can be used to promote a better and broader understanding of this phenomenon.

## Low silica concentrations are not necessarily “insignificant”

Recent studies on non-grass taxa have revealed that the dichotomous view of angiosperm taxa as being either high silica accumulators (e.g., grasses, other commelinids, and Cucurbitaceae) or low silica accumulators (i.e., most non-commelinid angiosperms) is flawed. First, some dicotyledonous species (other than those belonging to the Cucurbitaceae; e.g., *Abies pectinata, Cajanus cajan, Fagus sylvatica*, and *Helianthus annuus*) have silica concentrations which are as high or almost as high as those found in grasses (e.g., Hodson et al., 2005; Piperno, 2006). Second, there appear to be fundamental differences in the type, magnitude, patterns and functions of silicification between the high silica accumulating grasses and the low silica accumulating non-grass taxa. In grasses, it is generally accepted that silicification increases under higher silicon and water availability (reviewed in Katz et al., 2013) or if plants are exposed to prolonged herbivory (e.g., Soininen et al., 2013). In comparison, the effects of silicon and water availability on silicon accumulation in non-grass taxa are less clear. Cooke and Leishman (2012) found that silicification in non-grass species is probably less dependent on soil silica availability compared to silicification in grasses. Euliss et al. (2005) and Katz et al. (2013) found that the effects of water availability on silicification in non-grass species are weaker than in grasses growing under otherwise similar conditions. Herbivory seems to promote silicification more commonly in grasses compared to southwest Asian Asteraceae species, and it is thus also probable that silica plays less of a defensive role in Asteraceae species (Katz et al., 2014). Yet, even in plant species with very low silica concentrations, silica may play substantial roles, including reducing aluminum toxicity (Hodson and Evans, 1995; Britez et al., 2002; Khandekar and Leisner, 2011) and defending plants from pathogens (Fauteux et al., 2006; Zellner et al., 2011). Thus, low silica accumulation is not indicative of low silica function, and therefore silica function is not restricted to high silica grass taxa.

## Different families, different variability

There is both inter- and intra-familial variation in silica accumulation in plants. The grass family (approximately 10,000 species) and other commelinid families consist of high silica accumulators in which silicification patterns and roles are highly consistent. The effects of water availability (reviewed in Katz et al., 2013) and herbivory (Soininen et al., 2013) on silicification, as well as the multiple roles silica plays in grasses (Richmond and Sussman, 2003; Epstein, 2009; Cooke and Leishman, 2011; Guntzer et al., 2012), serve as good examples. The Orchidaceae (22,000-26,000 species) is a non-commelinid monocotyledonous family that is the most closely related to the commelinids (Bremer, 2000). The more ancient orchid subfamilies, Apostasioideaea and Cypripedioideae, are characterized by high silica contents, while members of the more derived Vanilloideae and Orchidoideae, and some Epidendrioideae species, have very low silica concentrations (Prychid et al., 2004). The loss of this trait, most notably in parallel to the transition from epiphytism to land-dwelling, is an evolutionary change that deserves more attention.

The Cucurbitaceae (1000 species) is a dicotyledonous family of high silica accumulators, but given there are dissimilarities in silicification mechanisms, patterns and roles between the two families it is likely that high levels of silicon accumulation evolved independently in this family (e.g., Mitani and Ma, 2005). The Asteraceae and Fabaceae, two of the largest angiosperm families (23,000 and 20,000 species, respectively) also demonstrate wide ranges of silica concentrations (Hodson et al., 2005; Piperno, 2006; Katz et al., 2013, 2014). At least in the case of the Asteraceae, it is now known that silicification patterns are probably poorly dependent on intra-familial phylogeny and soil silica concentrations (Katz et al., 2013, 2014). In other angiosperm families, such as the Rubiaceae (13,000 species), most studied species were shown to have very low silica concentrations (Piperno, 2006). Thus, there seem to be interesting patterns of silicification in families other than Poaceae which are likely to demonstrate variation in silica function and importance.

## Concluding remarks

It is clear that some groups, such as the grasses, commelinids and some aquatic macrophytes (Schoelynck et al., 2012), evolved the ability to accumulate silicon as herbivore defenses and a mechanism of alleviating abiotic stresses (e.g., Strömberg, 2011). Yet, these taxa represent only a part of the overall diversity of silicification among angiosperms. The inter-familial variability among non-grass taxa suggests that silicification is an ecologically-important trait in some of these taxa as well, especially in taxa which show silicification patterns resembling those found in grasses. Further studies of silica contents, silicification patterns and roles of silica in non-grass taxa are likely to elucidate physiological, ecological and evolutionary processes underlying the inter- and intra-familial variability in silica accumulation. This has the potential to increase our understanding of silicification and its significance in the biology and ecology of many species at a faster rate than through the study of Poaceae alone.

### Conflict of interest statement

The author declares that the research was conducted in the absence of any commercial or financial relationships that could be construed as a potential conflict of interest.
